# Impact of Treatment Supporters on the Treatment Outcomes of Drug Resistant-Tuberculosis (DR-TB) Patients: A Retrospective Cohort Study

**DOI:** 10.7759/cureus.22886

**Published:** 2022-03-06

**Authors:** Om P Giri, Abhay Kumar, Vishal P Giri, Nishant Nikhil

**Affiliations:** 1 Pulmonary Medicine, Darbhanga Medical College, Darbhanga, IND; 2 ENT, Uttar Pradesh University of Medical Sciences, Saifai, IND; 3 Pharmacology, Autonomous State Medical College, Shahjahanpur, IND; 4 Drug Resistant-Tuberculosis (DR-TB) Centre, Darbhanga Medical College, Darbhanga, IND

**Keywords:** multi-drug resistant tb -mdr, drug-resistant tuberculosis, tuberculosis treatment supporters, treatment outcome, dr-tb

## Abstract

Background: Drug resistant-tuberculosis (DR-TB) patients are provided universal drug susceptibility testing (UDST), anti-TB drugs for the treatment of DR-TB, nutritional support (Nikshay Poshan Yojana - the financial incentive of rupees five hundred per month for each notified DR-TB patient for the duration for which the patient is on anti-TB drugs) by the Government of India.

Methods: This retrospective cohort record-based study was conducted in DR-TB patients. Some 1095 DR-TB patients who have initiated treatment at Nodal DR-TB Centre, Darbhanga Medical College and Hospital (DMCH), Darbhanga, and continued their anti-TB drugs at home blocks were followed till their treatment outcome was known. Data were analyzed by statistical experts of DMCH.

Results: Treatment supporters comprised 688 (62.83%) females and 407 (37.17%) males. Different types of treatment supporters noted were accredited social health activists (ASHAs) 622 (56.80%), family members 365 (33.33%), and community health workers 108 (09.86%). Treatment outcome as transfer out was observed in 08 (1.29%), 10 (2.74%), and 13 (12.03%) cases among ASHAs, family members, and community health workers, respectively [statistically significant (p < 0.0001)].

Conclusion: ASHAs proved to be the best treatment supporters in comparison to both family members and community health workers for multi-drug resistant TB (MDR/RR-TB) patients.

## Introduction

Globally about half a million new cases of rifampicin resistant tuberculosis (RR-TB) occurred in 2019, out of which 78% had multi-drug resistant TB (MDR-TB). The estimated number of MDR/RR-TB cases in India is 124000 (9.1 per lakh population).

The DR-TB cases have been classified as (a) RR-TB (a TB patient, whose biological specimen is resistant to rifampicin); (b) MDR-TB (a TB patient, whose biological specimen is resistant to both isoniazid and rifampicin); (c) pre-extensively drug resistant (XDR)-TB (an MDR-TB patient with additional resistance to fluoroquinolone; (d) XDR-TB (pre-XDR-TB patient with additional resistance to either bedaquiline (Bdq) or linezolid (or both); and (e) H mono/poly DR-TB (a TB patient, whose biological specimen is sensitive to rifampicin but resistant to isoniazid).

At present the various DR-TB treatment regimens used for the management of DR-TB cases are (a) shorter oral Bdq-containing MDR/RR-TB regimen: it consists of intensive phase (IP) of [(4-6) Bdq (6), levofloxacin (Lfx), clofazimine (Cfz), Z, E, H-h, ethionamide (Eto)] and continuation phase (CP) of [5 (Lfx, Cfz, Z, E)], total duration is of 9-11 months ; (b) longer oral M/XDR-TB regimen is of 18-20 months with no IP and CP, [18-20 Lfx, Bdq (six months or longer), linezolid (Lzd), Cfz, cycloserine (Cs)]; (c) bedaquiline, pretomanid, and linezolid (BPaL) regimen [(6-9 months) BPaL] and (d) H mono/ poly DR-TB regimen [(six or nine months) Lfx, R, E, Z].

Shorter oral Bdq containing MDR/ RR-TB regimen: inclusion criteria are (a) MDR/ RR-TB with H resistance (INA/ KatG mutation only, not both), (b) MDR/ RR-TB with fluoroquinlone (FQ) resistance not detected, (c) children (aged 5 to less than 18 years age) and weighing at least 15 kg, (d) no history of exposure to Bdq, Lfx, ethionamide (Eto) or Cfz for more than one month, (e) no bilateral cavitatory disease/ extensive parenchymal damage, on chest radiography), (f) no military TB, TB meningitis, CNS-TB, (g) women who are not pregnant or lactating, (h) no uncontrolled cardiac arrhythmia, (i) QTcF interval less than 500 ms in electrocardiogram (ECG), (j) normal serum electrolytes, and (k) no history of additional risk factors for torsades de pointes. All the above criteria must be met.

Longer oral M/XDR-TB regimen: inclusion criteria are (a) MDR/ RR-TB patients who are excluded from shorter oral Bdq-containing regimen, (b) XDR-TB patients, and (c) additional resistance/intolerance/ non-availability of any drug used in shorter oral Bdq-containing regimen. Bedaquiline, pretomanid, and linezolid (BPaL) regimen is used in MDR-TB with additional resistance to FQ (pre-XDR-TB). H mono/ poly DR-TB regimen is used in H mono/poly DR-TB cases.

Stake holders of DR-TB services in local health system are physician at N/ DDR TBC, health facility doctor, DTO (District TB officer)/CDO (communicable disease officer), STS (senior treatment supervisor), STLS (senior TB laboratory supervisor), senior DR-TB co-ordinator, counselor at NDR-TBC, and treatment supporter. Each has a distinct function. Treatment supporter takes ambulatory care of the patient (counseling, linkages, support, and monitoring) [1]. 

The main role of a treatment supporter is to ensure that the patient takes the anti-TB drugs regularly, on schedule, for the full duration of treatment. The other roles of a treatment supporter are -- (a) to listen to the patient and encourage the patient to ask the questions about things that might be difficult to understand, (b) agree on a time and place to meet with the TB patient, (c) give the patient anti-TB drugs at each appointment according to the schedule, (d) record on the TB treatment card each time patient takes the drugs, and (e) refer the patient to the health facility when needed [2].

A treatment supporter can be any personnel from the medical officer, ANM (auxiliary nurse midwife), ASHA (accredited social health activist), CHW (community health worker), family member, and worker from the private sector (Project JEET). As far as possible only non-govt salaried personnel should be assigned as a treatment supporter to the patient. In exceptional circumstances, salaried National Tuberculosis Elimination Program (NTEP)/General Health System staff may also be assigned as a treatment supporter but they will not be eligible for any honorarium. Honorarium to the treatment supporter to be disbursed upon completion or cure of DR-TB patient is Rs. 5000 (five thousand). At the end of the intensive phase (IP) Rs. 2000 (Two thousand) or less - initiation date + six months has passed. At the end of the continuation phase (CP) Rs. 3000 (three thousand) or less -- if the patient has treatment outcome assigned as either “Cured” or “Treatment Completed” [3].

The present study aims to evaluate the impact of different types of TB treatment supporters on the final treatment outcomes of MDR/RR-TB patients.

## Materials and methods

Study design

The present study is a retrospective cohort study wherein, the details of the patients who underwent treatment were obtained from available hospital records. The institutional ethics committee approved the study and granted a waiver of the patient consent process.

Inclusion criteria

All MDR/RR-pulmonary TB patients, whose treatment was initiated at Nodal DR-TB Centre (NDR-TBC), Darbhanga Medical College & Hospital (DMCH), Laheriasarai, Darbhanga during the period from 1st September 2016 to 31st December 2020 and sent back through CDO (DTO) Darbhanga to their respective primary health centers (PHCs) in Darbhanga District were studied till their final treatment outcome was known.

Diagnosis and follow-up monitoring

Microbiological investigations of these patients were done at the time of diagnosis and during follow-up at NTEP accredited Damien Foundation India Trust (DFIT) medical laboratory (DMCH Campus, Darbhanga) which is well equipped with a fluorescent microscope, CBNAAT/ True Nat, FL-LPA, SL-LPA, and MGIT Culture. Their clinical monitoring was done at PHCs and laboratory investigations at the time of initiation of therapy and during follow-up were done in DFIT and Department of Clinical Pathology, DMCH.

Organizational structure

Darbhanga district has 18 PHCs (Tardih, Singhwara, Gora Bauram, Alinagar, Benipur, Manigachhi, Ghanshyampur, Biraul, Darbhanga Sadar, Baheri, Bahadurpur, Keoti, Kusheshwar Ashthan Satighat, Hanuman Nagar, Jale, Kusheshwar Ashthan East, Hayaghat, and Kiratpur), one CDO, one DOTS-PLUS Supervisor, one STS, and three TB Health Visitor (TBHV). ASHA, community health workers, and family members of the patients were engaged as treatment supporters in the service of MDR/RR-TB patients.

Treatment outcome criteria

(a) Cured -- A DR-TB patient who completed treatment with evidence of bacteriological response and no evidence of treatment failure. (b) Treatment completed -- A DR-TB patient who completed treatment but whose treatment outcome does not meet the definition for cured or treatment failed. (c) Died -- A DR-TB patient who died during the course of treatment. (d) Treatment failed -- A DR-TB patient whose treatment regimen needs to be terminated or permanently changed to a new regimen option or treatment strategy. (e) Lost to follow-up -- A DR-TB patient whose treatment was interrupted for two consecutive months or more. (f) Transferred out -- A DR-TB patient who was transferred to another treatment unit.

Statistical analysis

Pearson Chi-square test of independence was used and significant cut value was 95%.

## Results

A total of 1095 MDR/ RR-TB patients were studied, out of which 690 (63.01 %) were males and 405 (36.99%) were females. The minimum age of the patient was 7 years and the maximum age was 87 years. The age group of 21-40 years was the most affected [622 (56.80%)], followed by 7-20 years [241 (22.01%)]. The age group of 80-87 years was least affected [4 (0.36%)] (Table 1).

**Table 1:** Age group and gender distribution among DR-TB patients. DR-TB, Drug resistant-tuberculosis

Age group (years)	Male n (%)	Female n (%)	Total n (%)
07-20	130 (53.94)	111 (46.06)	241 (22.01)
21-40	400 (64.30)	222 (35.69)	622 (56.80)
41-60	123 (72.35)	47 (27.65)	170 (15.52)
61-80	34 (58.62)	24 (41.38)	58 (5.30)
80-87	03 (75.00)	01 (25.00)	04 (0.36)
Total	690 (63.01)	405 (36.99)	1095 (100.00)

Some 346 (31.60%) patients were declared cured, while 202 (18.45%) had treatment outcome as treatment completed. Thus treatment success (cured plus treatment completed) noted among MDR/ RR-TB patients was 548 (50.05%). Treatment failure was noted in 175 (15.98%) (Table 2).

**Table 2:** Treatment outcomes of DR-TB patients. DR-TB, Drug resistant-tuberculosis

Year	Total number n (%)	Cured n (%)	Treatment completed n (%)	Died n (%)	Treatment failed n (%)	Lost to follow-up n (%)	Transferred out n (%)
2016	51 (100.00)	20 (39.21)	4 (7.84)	11 (21.57)	3(5.88)	10 (19.61)	3 (5.88)
2017	141 (100.00)	62 (43.97)	18 (12.76)	31 (21.98)	9 (6.38)	13 (9.22)	8 (5.67)
2018	184 (100.00)	71 (38.59)	15 (8.15)	26 (14.13)	30 (16.21)	39 (21.19)	3 (1.63)
2019	334 (100.00)	83 (24.85)	78 (23.35)	50 (14.97)	49 (17.66)	47 (14.07)	17 (5.09)
2020	385 (100.00)	110 (28.57)	87 (22.60)	70 (18.18)	74 (19.21)	44 (11.43)	--
Total	1095 (100.00)	346 (31.60)	202 (18.45)	188 (17.17)	175 (15.98)	153 (13.97)	31 (2.83)

Some 62.83% of treatment supporters comprised females and 37.17% males. Some 48.40% of the treatment supporters belonged to the age group of 31-40 years, while 32.51% had age > 40 years. Some 71.23% of family members who acted as treatment supporters had age > 40 years, while 59.97% of ASHAs belonged to the age group of 31-40 years (Table 3).

**Table 3:** Baseline characteristics of treatment supporters.

Category	Type of treatment supporters			
Gender	Total n (%)	ASHA n (%)	Family member n (%)	Community health worker n (%)
Male	407 (37.17)	00	322 (90.96)	75 (69.44)
Female	688 (62.83)	622 (100.00)	33 (9.04)	33 (30.56)
Total	1095	622	365	108
Age group (years)				
18-30	209 (19.09)	155 (24.42)	05 (1.37)	49 (45.32)
31-40	530 (48.40)	373 (59.97)	100 (27.40)	57 (52.78)
>40	356 (32.51)	94 (15.11)	260 (71.23)	02 (1.85)
Total	1095	622	365	108

The ASHAs were associated as treatment supporters with 33.28% of cases who had treatment outcome as cured. Some 30.68% and 25.00% cured cases had treatment supporters as family members and community health workers respectively. The MDR/ RR-TB cases whose treatment outcome was declared as treatment completed had ASHAs, family members, and community health workers as treatment supporters in 18.33%, 19.73%, and 14.81% cases respectively. Treatment failure was observed in 15.91%, 15.89%, and 16.67% cases that were supported by ASHAs, family members, and community health workers, respectively. Death was recorded in 16.27% of cases in the family members group, 14.81% lost to follow-up cases were noted in the community health worker group (Table 4).

**Table 4:** DR-TB treatment outcomes in relation to different types of treatment supporters. DR-TB, drug resistant-tuberculosis; ASHA, accredited social health activists

Category	ASHA	Family member	Community health worker	Statistical indicator
Cured n (%)	207 (33.28)	112 (30.68)	27 (25.00)	χ2 =3.13, df=2, p=0.2
Treatment completed n (%)	114 (18.33)	72 (19.73)	16 (14.81)	χ2 =2.4, df=2, p=0.28
Died n (%)	107 (17.20)	63 (17.26)	18 (16.67)	χ2 =0.02, df=2, p=0.98
Treatment failed n (%)	99 (15.91)	58 (15.89)	18 (16.67)	χ2 =1.06, df=2, p=0.58
Lost to follow up n (%)	87 (13.90)	50 (13.70)	16 (14.81)	χ2 =0.086, df=2, p=0.95
Transferred out n (%)	08 (1.29)	10 (2.74)	13 (12.03)	χ2 =38.6, df=2, p<0.0001
Total	622	365	108	1095

Among DR-TB, the least transfer of patients was seen among ASHAs (1.29%) followed by family members (2.74%) and community health workers (12.03%) and this was statistically significant (p < 0.0001) (Table 4).

## Discussion

The present study revealed that males comprised 63.01% and females 36.99% of DR-TB cases. Some 56.80% of the DR-TB cases were of young age (21-40 years). This result is in accordance with a study conducted in Lucknow, which also ascertains that the males (69.40%) suffer from DR-TB more than females (30.60%) and the age group most involved was 21-30 years. Males were predominant (54.00%) but higher age group (25-64 years) as the most affected (74.00%) has been recorded by another study conducted in the Netherlands. Female predominance (56.10%) among DR-TB cases has been reported by Javaid et al., and this gender distribution is not in accordance with our study and few studies mentioned above as all of them have mentioned males predominance in DR-TB [4-6].

The present study revealed that a larger number (62.83%) of treatment supporters comprised females as compared to the males (37.17%). Female treatment supporters comprised 622 (56.80%) ASHAs, 33 (3.01%) family members, and 33 (3.01%) community health workers. Most (48.40%) treatment supporters belonged to the age group of 31-40 years, followed by (32.51%) more than 40 years age group. Family members of more than 40 years and ASHAs of 31-40 years were preferred as treatment supporters.

Females predominance (57.82%) as treatment supporters has been also reported by a retrospective cohort study in Pakistan. Females comprised 60.00% family supporters, 51.10% health facility supporters, and 37.20% community supporters but most (41.39%) treatment supporters had different age groups (15-25 years) as compared to the age groups (31-40 years) noted in the present study. This study further revealed that family members were chosen as treatment supporters by 86.80% of patients, 7.63% of patients selected community-based health workers, and 5.56% of cases chose facility-based health workers. Thus, family members were noted to be the first choice as treatment supporters. Our study is not in accordance with this study report as the present study has revealed that family members were chosen by 33.33% of patients (second choice), 9.86% of cases selected community-based health workers, and 56.80% cases chose a facility-based health worker as treatment supporters [7].

Family members have also been reported as the second most preferred (38.36%) type of treatment supporters in another cross-sectional study in Pakistan and a qualitative study in Nigeria noticed maximum emotional and physical support from the members of the family. The community-based treatment provider has been reported as the first choice (71.60%) treatment provider in a mixed-method study in Uganda [8-11]. The present retrospective study revealed ASHAs as the most preferred treatment supporters by the patients, while family members were their second choice; a similar observation has also been made by Alipanah et al. [12].

The present study demonstrated that cure, treatment completion, death, treatment failure, or loss to follow-up were not affected by the type of treatment supporter but has an impact on transfer out. Another study has demonstrated that treatment outcome is not affected by the type of treatment supporter, so a patient should be allowed to choose a treatment supporter of choice [4].

A study conducted in Swaziland revealed the significant impact of treatment supporters on treatment outcomes as treatment completed, died, and transferred out but the significant impact was noticed on treatment outcomes as cured, treatment failed and defaulted. Some 60.90% having treatment supporters completed the treatment, 10.60% had died, and only 2.5% cases were transferred out. Some studies have demonstrated a significantly higher treatment success rate among DR-TB patients supported by treatment supporters. A cross-sectional survey in Rawalpindi revealed different success rates and transferred out cases with different types of treatment supporters. Treatment success rates with female health workers, community health workers, and family members recorded were 93.10%, 89.00%, and 73.50% respectively, while transferred out cases noted were 2.0%, 00.00%, and 3.20% respectively [13-16].

The present study revealed some impact of different types of treatment supporters on the treatment outcome. The significant impact of treatment supporters on treatment outcome as transferred out has been noted, while no significant impact of treatment supporters on treatment outcomes as cured, treatment completed, died, treatment failed, and lost to follow up has been demonstrated in the present study.

Transferred out occur when patients are not satisfied with the support provided by the treatment providers, Patients also try to migrate from their native place in Bihar to other states of India for jobs, business, and higher study. Counseling plays an important role in convincing them not to move outside the state till their treatment is completed. Some patients migrate to urban centers from rural ones with the hope to get better treatment facilities. It may also be due to the fact that treatment supporters start taking less interest in their work for not getting their honorarium in time [17].

## Conclusions

The present study provides an assessment of different treatment supporters in the programmatic management of drug resistant tuberculosis (PMDT) program in India. This study highlights the role of different support providers and the impact of different types of treatment supporters on the treatment outcome of DR-TB patients. A significant impact of some treatment supporters is also evident in the treatment outcome. ASHAs seem to play a very important role as treatment supporters of DR-TB patients by decreasing or preventing the proportion of transfer out cases and this could be due to training that they have received and good counseling by them right from the start of the treatment.
